# Predictive value of blood routine-derived inflammation indexes for varicella-zoster virus meningitis in patients with acute herpes zoster

**DOI:** 10.3389/fcimb.2025.1617460

**Published:** 2025-06-24

**Authors:** Yan Zhang, Yanrong Yuan, Huili Liu, Jun Wang, Yongxing Yan

**Affiliations:** Department of Neurology, Hangzhou Third People’s Hospital, Hangzhou, China

**Keywords:** varicella-zoster virus, herpes zoster, meningitis, inflammation, platelet-to-lymphocyte ratio

## Abstract

**Background:**

Blood routine-derived inflammation indexes have been studied in many diseases. However, their roles in varicella-zoster virus (VZV)-related central nervous system (CNS) infection diseases are unclear. This study analyzed the value of blood routine-derived inflammatory indexes in the diagnosis and prognosis of VZV meningitis in patients with acute herpes zoster (HZ).

**Methods:**

A total of 31 cases of VZV meningitis were selected as the research subjects, with 64 cases of acute HZ admitted to our hospital during the same period as the control group. The blood routine markers and blood routine-derived inflammatory indexes were compared between the two groups. Univariate analysis, logistic regression, and receiver operating characteristic (ROC) curve analysis were used to evaluate the correlation between the levels of each inflammatory marker and the occurrence of VZV meningitis, as well as to evaluate the relationship between the inflammatory markers and the prognosis of VZV meningitis at discharge.

**Results:**

Compared with those in the HZ group, the patients in the VZV meningitis group showed significantly increased platelet-to-lymphocyte ratios (PLRs), neutrophil-to-lymphocyte ratios (NLRs), and systemic immune-inflammation index (SII) scores (*p* < 0.05 and *p* < 0.01), while the absolute counts of lymphocytes decreased (*p* < 0.05). Multivariate logistic regression analysis showed a correlation between the PLR and the occurrence of VZV meningitis. The PLR also showed a significant correlation with the prognosis of VZV meningitis patients at discharge (*r* = 0.774, *p* < 0.01).

**Conclusion:**

The PLR, as a potential inflammatory index, may have certain clinical value in predicting the occurrence and prognosis of VZV meningitis.

## 1 Introduction

Varicella-zoster virus (VZV) is a DNA virus with humans as its only natural host. It can be transmitted through droplets and/or contact. The primary infection mainly causes chicken pox. Residual VZV can travel retrograde along the sensory nerve axons or through the fusion of infected T cells and neuronal cells, transfer to the posterior root ganglia or cranial ganglia of the spinal cord, and lurk. When the host’s resistance decreases, the VZV-specific cellular immunity decreases and the latent virus can be reactivated, replicating in large quantities, then transferring to the skin through the sensory nerve axons, penetrating the epidermis, and causing herpes zoster (HZ) (Sauerbrei, 2016). Relevant studies have shown that the incidence of HZ is increasing year by year (Harpaz and Leung, 2019; Soysal et al., 2021; van Oorschot et al., 2021). From 2011 to 2019, the incidence of HZ in Turkey was 1.82–2.85/1,000 person-years, showing a slow upward trend (Soysal et al., 2021). This growth phenomenon was also found in the United States (Harpaz and Leung, 2019). In China, there are some differences in the incidence rates of HZ in different regions, but they are also increasing with each passing year (Li et al., 2016; Lu et al., 2018).

Although most patients with acute HZ can fully recover after treatment, there are still many patients who may have a series of complications. A number of patients may experience complications of central nervous system (CNS) infections, among them meningitis being a common CNS complication. Previous studies have also found that VZV is one of the most common pathogens of aseptic meningitis (Becerra et al., 2013). Once CNS infection occurs in patients with HZ, this will prolong their hospitalization time, affect their quality of life, increase the economic burden on both patients and society, and, in severe cases, even endanger their lives. The clinical manifestations of VZV meningitis are diverse and lack specificity. They may present with clinical symptoms such as fever, headache, nausea and vomiting, and cognitive impairment, among others. However, most patients with VZV meningitis have good prognosis after early active treatment, even without any sequelae. Therefore, early diagnosis and treatment of VZV meningitis are extremely important. At present, cerebrospinal fluid (CSF) testing has significant value for the diagnosis of VZV meningitis, of which VZV DNA positivity in the CSF is the gold standard. However, obtaining CSF samples is relatively difficult. For clinicians, it is essential to determine more easily obtainable objective indicators for the early diagnosis of VZV meningitis. Of these, blood biomarkers are the preferred choice.

Previous studies found that the pathogenesis of VZV-related CNS infection may be associated with viral infection, inflammatory response, and vasculitis, among others. Currently, many scholars believe that vasculitis is the most likely cause (Hung et al., 2012; Nagel et al., 2020; Yan et al., 2022). However, a study analyzed the clinical characteristics and prognosis of 123 patients with VZV meningitis confirmed by positive VZV DNA in the CSF and compared these with those of 12 patients with VZV encephalitis. The leukocyte counts in the CSF of VZV meningitis were significantly higher than those of VZV encephalitis. Brain magnetic resonance imaging (MRI) of VZV meningitis showed no abnormalities, while imaging of the patients with VZV encephalitis showed vasculitis in 50% of cases. It is believed that the pathogenesis of VZV meningitis might be different from that of VZV encephalitis, with the pathogenesis of VZV meningitis being more related to the host’s inflammatory response (Dulin et al., 2024). Our study on CSF proteomics in patients with VZV-related meningitis found that the expression levels of the proteins that reflect inflammation activation, such as IL-1RN, MPO, CXCL10, and PRTN3, were significantly increased (Liu et al., 2023). Ramachandran et al. (2021) found that 10 typical inflammatory markers, namely, IL-6, IL-8, IL-10, IL-17F, IL-1RA, interferon R, CXCL-9, CXCL-10, CCL-2, and G-CSF, were significantly elevated in the CSF of patients with VZV meningitis. This further suggests that the inflammatory response may be an important pathophysiological mechanism of VZV meningitis. Therefore, we hypothesized that the level of blood inflammatory markers may be beneficial for the early diagnosis of VZV meningitis.

The measurement of blood routine is convenient and fast, and patients can obtain the results quickly after outpatient testing. In blood routine markers, there are both single inflammatory indicators and combined “inflammation indexes” that can be calculated through multiple single indicators. Among them, the platelet/lymphocyte ratio (PLR), the neutrophil/lymphocyte ratio (NLR), the neutrophil/platelet ratio (NPR), and the systemic immune inflammation index (SII) are the commonly used derived inflammation indexes. They can not only predict infectious diseases but also play a crucial role in the assessment of the prognosis of diseases, with their effect being better than that of a single indicator. Therefore, this study analyzed the value of the blood routine-derived inflammation indexes for the diagnosis and prognosis of VZV meningitis, which can provide a reference for clinicians for early identification and intervention.

## 2 Patients and methods

### 2.1 Patients and grouping

A retrospective study was conducted on 31 patients with VZV meningitis, diagnosed by positive VZV DNA in the CSF, who were admitted to our hospital from January 2021 to December 2023. The inclusion criteria were: 1) diagnosis of VZV meningitis based on the criteria in our previous study (Yan et al., 2022) and meets the criteria of the European consensus-based guidelines on the management of HZ (Akya et al., 2015); 2) age over 14 years; and 3) complete clinical data (e.g., symptoms, signs, auxiliary examinations, and treatment). The following were excluded: 1) patients with incomplete clinical records; 2) those with zoster sine herpete; 3) breastfeeding or pregnant women; and 4) individuals with tumor or with HIV infection.

A total of 64 patients with acute HZ who were hospitalized during the same period and who were matched for age, gender, and comorbidities with the VZV meningitis group were selected as the control group. The inclusion criteria were as follows: 1) all of the patients’ clinical symptoms, signs, and auxiliary examinations meet the diagnostic criteria for HZ (Akya et al., 2015), with the diagnosis being determined by a dermatologist; 2) within 2 weeks of onset and without relevant treatment; 3) age of over 14 years; and 4) complete clinical data. The exclusion criteria were the same as those for the VZV meningitis group.

This study was approved by the Ethics Committee of Hangzhou Third People’s Hospital (no. 2021KA013). All procedures were conducted in accordance with the Helsinki Declaration.

### 2.2 Methods

#### 2.2.1 Data collection

The clinical data of these patients were collected through the hospital’s clinical electronic medical record system and the hospital information system, which included: 1) demographic characteristics and disease-related information, e.g., gender, age, comorbidities, disease duration, herpes location, and herpes treatment status, and 2) laboratory blood routine test results at admission, e.g., red blood cell counts, hemoglobin, platelet counts, white blood cell counts, neutrophil counts, and lymphocyte counts, as well as the NLR (calculated as NLR = neutrophil count/lymphocyte count), the PLR (calculated as PLR = platelet count/lymphocyte count), the NPR (calculated as NPR = neutrophil count/platelet count), and the SII (calculated as SII = platelet count × neutrophil count/lymphocyte count).

#### 2.2.2 Prognostic assessment of VZV meningitis patients at discharge

The prognosis of patients with VZV meningitis was evaluated at discharge using the modified Rankin Scale (mRS). The mRS ranges from level 0 to level 6, with each level representing a different degree of neurological recovery and disability status of the patient.

Level 0: The patient has no obvious symptoms, has fully recovered, or is close to normal neurological function.
Level 1: The patient has mild sequelae, but no obvious functional impairment. Patients can complete all daily responsibilities and activities, and their daily work and life are basically not affected.
Level 2: The patient has mild sequelae and is restricted on some daily activities, but can handle personal affairs without the need for assistance.
Level 3: The patient is moderately disabled and requires some assistance to complete daily activities, but can walk independently.
Level 4: The patient has severe disability sequelae and cannot walk independently, requiring assistance in daily life.
Level 5: The patient has severe disabilities, requires bed rest and is incontinent, and requires continuous care and attention.
Level 6: The patient has died.

The mRS scores of each patient at discharge were established unblinded by consensus of two authors (neurologists), with levels 0–2 indicating a favorable prognosis and levels greater than 2 indicating an unfavorable prognosis.

#### 2.2.3 Statistical analysis

SPSS 17.0 statistical software was used for data processing. Count data are expressed in numbers (percentage). Comparisons between groups were performed using the chi square or Fisher’s exact method. The measurement data conform to a normal distribution and are represented as the mean ± standard deviation (*X* ± SD). Comparisons between two groups were conducted using a grouped *t-*test. Multiple logistic regression analysis was used to investigate the influencing factors of HZ complicated with meningitis. Before the logistic regression analysis, the multicollinearity among the PLR, NLR, SII, and lymphocyte counts was evaluated by calculating the variance inflation factor (VIF). Receiver operating characteristic (ROC) curve analysis was used to evaluate the value of PLR in predicting meningitis in patients with HZ. Given that the mRS score is an ordinal variable, we used Spearman’s rank correlation analysis to evaluate the correlation between the inflammatory markers and the prognosis of patients with VZV meningitis. The difference is statistically significant with *p* < 0.05.

## 3 Results

### 3.1 Baseline characteristics of the two groups

Among the 31 patients with VZV meningitis, there were 19 men and 12 women, aged 21–70 years (55.2 ± 15.6 years). Of the 64 patients in the HZ group, 29 were men and 35 women, aged 22–80 years (56.7 ± 14.8 years). There were no significant differences between the two groups in terms of age, sex, and comorbidity [hypertension, diabetes, coronary heart disease, stroke, chronic obstructive pulmonary disease (COPD), chronic kidney disease, autoimmune disease, etc.] (*p* > 0.05) (**Table 1**).

**Table 1:** Baseline characteristics of patients in different groups at admission.

Characteristics	VZV meningitis group (*n* = 31)	Herpes zoster group (*n* = 64)	*t*/*χ* ^2^	*p*
Age (mean ± SD) (years)	55.2 ± 15.6	56.7 ± 14.8	0.453	0.651
Gender				
Men, *n* (%)	19 (61.3)	29 (45.3)	2.133	0.144
Women, *n* (%)	12 (38.7)	35 (54.7)		
Comorbidity (cases)				
Coronary heart disease	0	4	NA	NA
COPD	0	2	NA	NA
Hypertension	12	19	0.773	0.379
Immune diseases	3	2	1.798	0.180
Stroke	1	2	0.001	0.979
Diabetes	2	9	1.182	0.277
Chronic kidney disease	0	1	NA	NA
Herpes zoster site, *n*				
Head and neck	21	30	6.277	0.099
Chest and back	7	13		
Waist and abdomen	2	11		
Limb	1	10		

*VZV*, varicella zoster virus; *COPD*, chronic obstructive pulmonary disease; *NA*, not applicable.

In the VZV meningitis group, 21 cases had herpes lesions in the head and neck, seven cases in the chest and back, two cases in the waist and abdomen, and one case in the limbs. In the HZ group, 30 cases had herpes lesions in the head and neck, 13 cases in the chest and back, 11 cases in the waist and abdomen, and 10 cases in the limbs. There was no significant difference in the location of herpes between the two groups (*p* > 0.05) (**Table 1**).

### 3.2 Comparison of the inflammatory index levels between the two groups of patients at admission

Compared with that in the HZ group, the PLR (194.0 ± 111.8 *vs*. 127.0 ± 40.0), NLR (3.61 ± 2.10 *vs*. 2.70 ± 1.72), and SII (790.7 ± 467.6 *vs*. 526.4 ± 281.4) in the VZV meningitis group were significantly increased (*p* < 0.05 and *p* < 0.01). The lymphocyte counts (1.43 ± 0.74 *vs*. 1.73 ± 0.57) decreased (*p* < 0.05). There were no significant differences in the counts of white blood cells, red blood cells, platelets, and hemoglobin and in the NPR between the two groups (*p* > 0.05) (**Table 2**).

**Table 2:** Comparison of the inflammatory index levels between the two groups.

Characteristics	VZV meningitis group (*n* = 31)	Herpes zoster group (*n* = 64)	*t*	*p*
White blood cell (×10^9^/L)	6.26 ± 2.65	6.46 ± 2.04	0.414	0.680
Red blood cell (×10^12^/L)	4.25 ± 0.41	4.47 ± 0.58	1.886	0.063
Hemoglobin (g/L)	132.5 ± 13.0	136.6 ± 16.8	1.210	0.230
Platelet (×10^9^/L)	220.3 ± 43.0	201.7 ± 59.2	1.563	0.122
Lymphocyte count (×10^9^/L)	1.43 ± 0.74	1.73 ± 0.57	2.156	0.034
Neutrophil count (×10^9^/L)	4.27 ± 2.35	4.16 ± 1.65	0.261	0.795
PLR	194.0 ± 111.8	127.0 ± 40.0	4.278	0.000
NLR	3.61 ± 2.10	2.70 ± 1.72	2.253	0.027
NPR	0.02 ± 0.01	0.02 ± 0.01	0.985	0.327
SII	790.7 ± 467.6	526.4 ± 281.4	3.428	0.001

*VZV*, varicella zoster virus; *PLR*, platelet-to-lymphocyte ratio; *NLR*, neutrophil-to-lymphocyte ratio; *NPR*, neutrophil-to-platelet ratio; *SII*, systemic immune-inflammation index.

### 3.3 Multivariate logistic regression analysis

Using the presence of concurrent meningitis as the dependent variable, multivariate binary logistic regression analysis was conducted, with the statistically significant factors in the univariate analysis (PLR, NLR, SII, and lymphocyte counts) as independent variables. It was found that the PLR is an independent risk factor for meningitis in patients with HZ (*p* < 0.05), while the NLR, SII, and lymphocyte counts were not independent risk factors (*p* > 0.05) (**Table 3**).

**Table 3:** Multivariate logistic analysis of the risk factors of concomitant meningitis in patients with acute herpes zoster (HZ).

Variable	*β value*	SE value	Wald	OR value	95%CI	*p value*
Lymphocyte count	0.809	0.648	1.561	2.246	0.631–7.996	0.212
PLR	0.023	0.009	6.916	1.023	1.006–1.041	0.009
NLR	−0.138	0.256	0.291	0.871	0.528–1.438	0.590
SII	0.001	0.001	0.407	1.001	0.998–1.004	0.524

*PLR*, platelet-to-lymphocyte ratio; *NLR*, neutrophil-to-lymphocyte ratio; *SII*, systemic immune-inflammation index.

**Figure 1:**
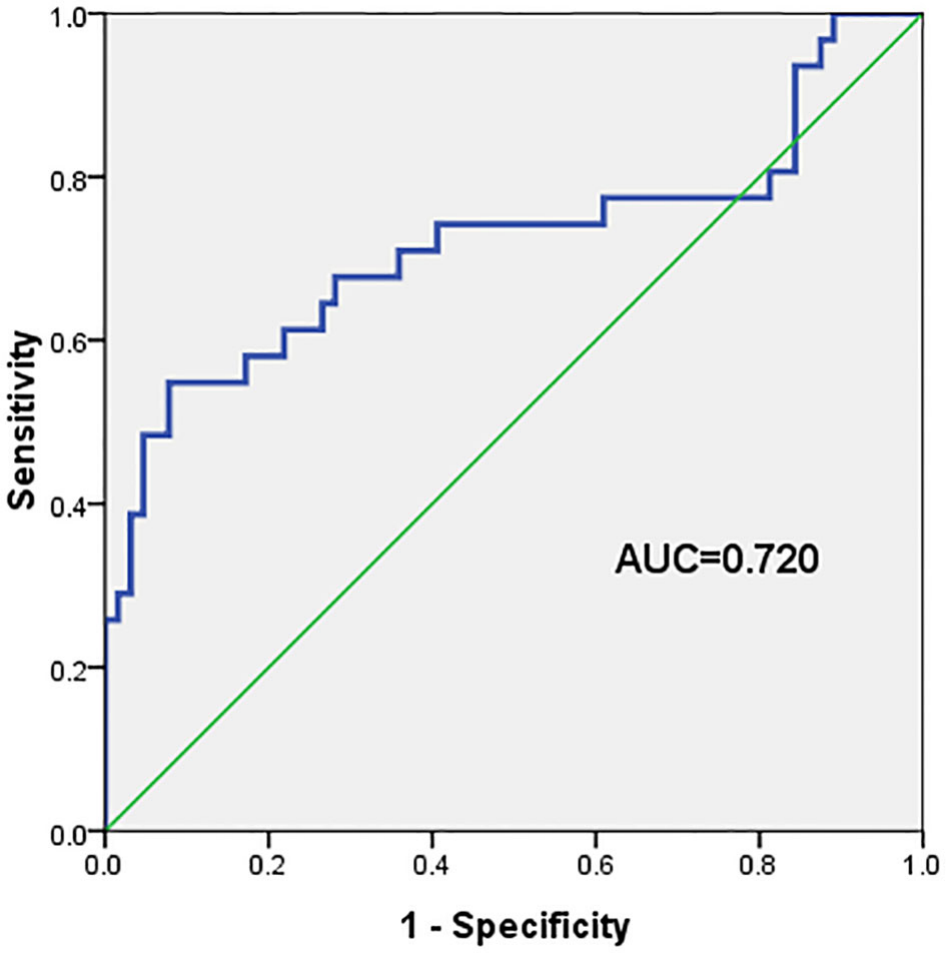
The area under the curve (AUC), sensitivity, and specificity of the platelet-to-lymphocyte ratio (PLR) in predicting the occurrence of varicella-zoster virus (VZV) meningitis in patients with acute herpes zoster were 0.720, 58.1%, and 82.8%, respectively.

The ROC curve for the prediction of VZV meningitis was established based on the PLR. The results showed the predictive effect of PLR on the occurrence of VZV meningitis, with area under the curve (AUC) = 0.720. The sensitivity and specificity were 58.1% and 82.8%, respectively (**Figure 1**).

### 3.4 Comparison of the inflammatory index levels in VZV meningitis patients with different prognosis

The mRS scores among the 31 VZV meningitis patients at discharge were as follows: 14 cases at level 0, nine cases at level 1, four cases at level 2, three cases at level 3, and one case at level 4. According to the prognostic criteria for VZV meningitis, 27 cases had a favorable prognosis, while four cases had an unfavorable prognosis. Compared with that in the group with favorable prognosis, the PLR in the group with unfavorable prognosis was significantly increased (*p* < 0.01). However, there were no significant differences in the other parameter such as age, gender, NLR, NPR, and SII (*p* > 0.05) (**Table 4**).

**Table 4:** Comparison of the inflammatory index levels in varicella-zoster virus (VZV) meningitis patients with different prognosis.

Characteristics	Favorable prognosis VZV meningitis group (*n* = 27)	Unfavorable prognosis VZV meningitis group (*n* = 4)	*t*/*χ* ^2^	*p*
Age (years), mean ± SD	56.1 ± 15.6	48.8 ± 16.5	0.878	0.387
Gender				
Men, *n* (%) Women, *n* (%)	18 (66.7) 9 (33.3)	1 (25.0) 3 (75.0)	2.549	0.110
White blood cell (×10^9^/L)	6.6 ± 2.7	4.1 ± 0.9	1.784	0.085
Red blood cell (×10^12^/L)	4.3 ± 0.4	4.0 ± 0.2	1.129	0.268
Hemoglobin (g/L)	132.6 ± 13.9	131.5 ± 4.8	0.160	0.874
Platelet count (×10^9^/L)	221.7 ± 41.8	215.3 ± 57.4	0.249	0.805
Lymphocyte count (×10^9^/L)	1.5 ± 0.7	0.8 ± 0.4	1.896	0.068
Neutrophil count (×10^9^/L)	4.5 ± 2.4	2.8 ± 1.0	1.399	0.172
PLR	172.4 ± 70.2	339.3 ± 223.5	3.181	0.004
NLR	3.6 ± 2.2	4.0 ± 1.8	0.385	0.703
NPR	0.02 ± 0.01	0.01 ± 0.003	1.426	0.165
SII	778.4 ± 481.3	873.4 ± 410.3	0.374	0.712

*PLR*, platelet-to-lymphocyte ratio; *NLR*, neutrophil-to-lymphocyte ratio; *NPR*, neutrophil-to-platelet ratio; *SII*, systemic immune-inflammation index.

### 3.5 Correlation analysis between the blood routine-derived inflammation indexes and the prognosis of VZV meningitis

**Figure 2:**
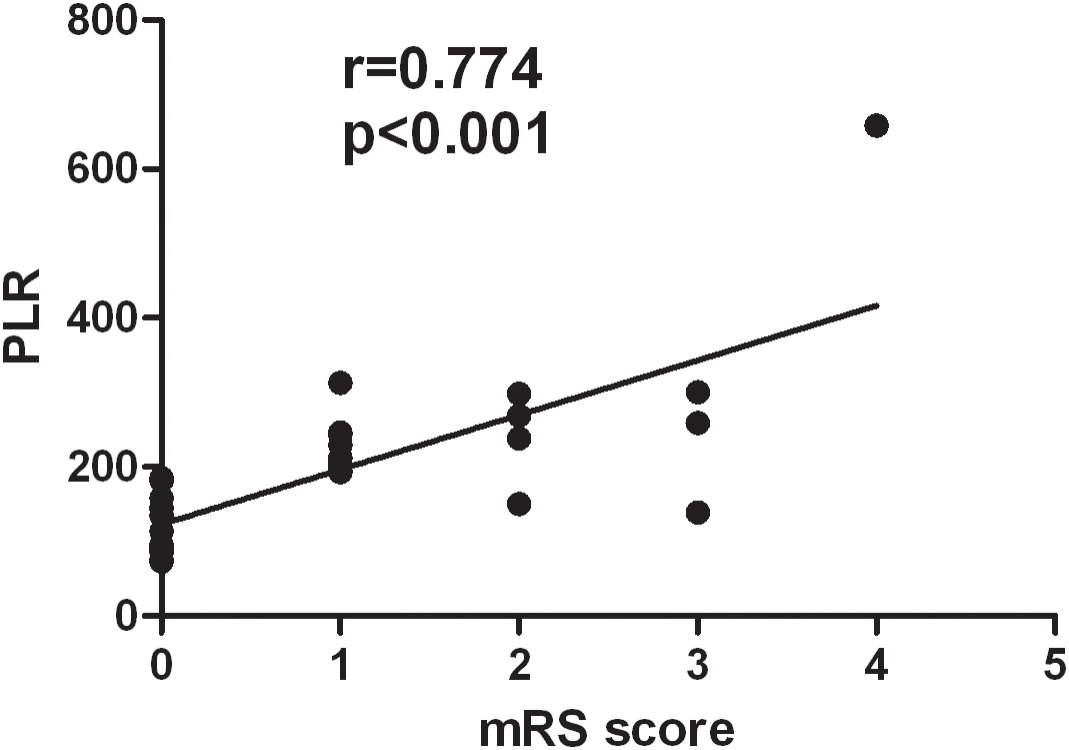
There was a positive correlation between the platelet-to-lymphocyte ratio (PLR) and the modified Rankin Scale (mRS) scores of patients with varicella-zoster virus (VZV) meningitis at discharge (*r* = 0.774, *p* < 0.001).

The correlation analysis showed that the PLR was positively correlated with the mRS scores of patients with VZV meningitis at discharge (*r* = 0.774, *p* < 0.001), as shown in **Figure 2**. There was no significant correlation (*p* > 0.05) between the mRS scores and other inflammatory indexes such as the NLR, NPR, and SII in patients with VZV meningitis.

## 4 Discussion

VZV, also known as human herpes virus 3 (HHV-3), belongs to the alpha herpes virus subfamily and has a double-stranded DNA (Ozdemir et al., 2000). VZV has nerve invasiveness. Under natural conditions, it only infects humans (Bhojwani et al., 2022). The initial infection of VZV in children can lead to chicken pox, and the virus remains dormant in the ganglia of the cranial nerves, dorsal roots, and autonomic nervous system for a long time. The reactivation of VZV can cause HZ (Kennedy and Mogensen, 2020). It can also cause CNS infections (Andrei and Snoeck, 2021; Werner et al., 2017). Epidemiological studies have shown that the incidence of VZV-related CNS infection is approximately 0.65–3.0 per 100,000 person-years (Arruti et al., 2017; Becerra et al., 2013). The relative incidence of CNS infection among patients with HZ ranged from 0.02% to 2.7% (Forbes et al., 2021; Giannelos et al., 2024; Schmidt et al., 2016), with a higher incidence of CNS infections being reported in HZ patients with immunocompromised comorbidities including HIV infection, stem cell transplantation, and solid organ and hematological malignancies (Blank et al., 2012; Blennow et al., 2014; Yenikomshian et al., 2015). The incidence of varicella has significantly decreased since the application of the VZV vaccine, but the complications of VZV-related CNS diseases are more common and diversified than previously thought (Gershon et al., 2015). Meningitis, encephalitis, cerebellitis, cerebral vasculitis, and myelitis are the main clinical phenotypes of VZV-related CNS infections. Previous studies have reported that meningitis is the most common type. For example, in a multicenter study on viral meningitis from Spain (Becerra et al., 2013), enterovirus (76.8%) was found as the most common pathogen, with VZV (10.3%) ranking second among the causes of viral meningitis. In the VZV-related CNS infection group collected in France, meningitis accounted for 58.3% and encephalitis accounted for 41.7% (Kennedy and Mogensen, 2020). Once HZ patients present with concurrent CNS infection, this not only increases their economic burden but also reduces their quality of life. Early diagnosis and treatment can improve patients’ quality of life.

The diagnosis of VZV meningitis is mainly based on clinical symptoms, imaging, and CSF characteristics. Of these, positive VZV DNA in the CSF is the gold standard for the diagnosis of VZV- related CNS infection. Positive VZV DNA was found in the CSF of the included patients in this study, and all showed herpes on the skin, ensuring the accuracy of patient inclusion. However, in the real-world clinic, some patients with HZ may only experience pain in the early stages and may not present with a rash, which can be easily misdiagnosed as, e.g., coronary heart disease, digestive tract disease, and cervical and lumbar spine disease (Kennedy and Mogensen, 2021), as well as acute abdomen, leading to surgical errors (Amjadi et al., 2017). The diagnosis of VZV-related CNS infection without rash manifestations in the early stages is more difficult. Therefore, it is extremely necessary to search for more easily accessible laboratory biomarkers for the early prediction of VZV meningitis.

At present, the mechanism of VZV meningitis is not fully understood. It may be related to inflammation and immune dysfunction when commonly recognized, but differs from that of VZV encephalitis (Dulin et al., 2024; Parkes-Smith and Chaudhuri, 2022). As is well known, neutrophils, lymphocytes, monocytes, and platelets can be utilized as experimental markers to reflect the inflammatory and immune status of the body. However, these single indicators vary and fluctuate greatly among different individuals and are influenced by multiple factors. By combining the ratios of the above indicators, individual differences and instability can be eliminated. These ratios also reflect the balance between the host inflammatory response and the immune status. Many research studies have shown that the combination of indicators such as NLR, PLR, and SII can more sensitively reflect the balance between the host’s inflammatory response and the immune status (Le Bot et al., 2021; Ozdemir et al., 2000).

The NLR is known as one of the best validated peripheral blood-derived inflammatory markers. It has been used in various diseases such as different types of cancers, in total of over 80,000 patients (Dolan et al., 2017). It has also been extensively used in non-cancerous conditions, including in the prognosis of patients with acute myocardial infarction (Zhang et al., 2018) and COPD (Sakurai et al., 2018), among others. It has been reported that an increased NLR may be an independent risk factor for diabetes peripheral neuropathy (DPN). When the NLR is higher than 2.13, the specificity and sensitivity of diagnosing DPN are 48.1% and 81.3%, respectively (Xu et al., 2017). Another study reported that an NLR of 7.3 was an effective cutoff to detect acute exacerbations of COPD (Farah et al., 2017). Kazancioglu et al. (2023) also reported that the NLR can be used as a biomarker to differentiate bacterial meningitis from viral meningitis and to predict the prognosis of CNS infection. However, it is difficult to establish an exact cutoff value. PLR is one of the biomarkers related to inflammation in the body, and as a composite indicator of inflammation, it is closely related to the immune inflammatory state. Platelets are subcellular fragments of megakaryocytes in the bone marrow, and they have been considered as an extension of the cellular immune system, playing a role in leukocyte functioning and the subsequent release of inflammatory signals. When platelets detect microorganisms through Toll-like receptors (TLRs), they are activated and can release many pro-inflammatory cytokines. These pro-inflammatory factors are recruiters and activators of leukocytes, playing important roles in the immune regulatory functions of platelets (Carestia et al., 2023).Platelet–neutrophil interactions increase the oxidative burst, phagocytosis, and neutrophil survival, playing an important role in the host inflammatory reaction (Wu et al., 2009). Disease and the effects of drugs can affect the production of platelets, and lymphocytes are responsible for immune monitoring and editing. Therefore, the PLR is often used for the diagnosis and prognostic evaluation of chronic inflammation, sepsis, and other diseases. A previous study used 262 as the cutoff value and found that the PLR had a sensitivity and specificity of 75% and 85.7%, respectively, in predicting the efficacy of systemic corticosteroid therapy in ulcerative colitis (Endo et al., 2021). Another study reported that, when the PLR cutoff value was 85.9, the sensitivity of detecting inflammatory bowel disease activity was better (93.6%) (Nassri et al., 2020). Some studies have suggested that the SII could also be used to predict the prognosis of patients with cancer (Zhong et al., 2017), vasculitis (Kim et al., 2019), and dementia (van der Willik et al., 2019), among others. This study analyzed the above indicators in patients with VZV meningitis. Compared with those in patients with HZ, the PLR, NLR, and SII in patients with VZV meningitis were significantly increased. The absolute counts of lymphocytes decreased. Further analysis revealed that the PLR has a good predictive effect on the occurrence of VZV meningitis. Based on the previous analysis, we believe that the increased PLR may reflect the intensity of the inflammatory response in patients with VZV meningitis and that it may be related to the platelets involved in the host inflammatory and immune response. Our research findings further indicated that patients with VZV meningitis exhibit significant inflammatory responses.

After early active and formal treatment, most patients with VZV meningitis have a good prognosis (Becerra et al., 2013; Grahn and Studahl, 2015). This study included 31 patients with VZV meningitis. The favorable prognosis at discharge was 87.1% (27/31), which is similar to that in previous reports. At present, there are differences in the criteria for evaluating prognosis, with most of them using the Glasgow Outcome Scale (GOS) for assessment. However, the GOS is mostly used for predicting the prognosis of patients with severe brain injury. VZV meningitis patients have no symptoms of brain parenchymal damage, and their symptoms are milder compared with those of encephalitis patients. Using the GOS to determine the prognosis of VZV meningitis patients may lead to bias. This study used the mRS to assess the severity of the neurological symptoms and to determine the prognosis of patients. It is an easy-to-use scale, which scores disability from 0 to 6 and describes patient functionality in general terms, and can quantify the neurological function status of patients, which helps doctors in developing treatment plans and predicting prognosis. It is also simple, practical, and has good reliability and authenticity. There is no physical examination-related content in the mRS, and remote evaluation can be achieved through telephone or questionnaire forms. It is suitable for evaluating the prognosis of various neurological diseases. Of course, the mRS also has some shortcomings, and using more objective laboratory markers to assess prognosis is of great value to clinicians. This study found a positive correlation between PLR and the prognosis of VZV meningitis at discharge, which may be an effective objective indicator for predicting prognosis and is beneficial for clinicians.

This study has several limitations. Firstly, due to the low incidence rate of VZV-related meningitis and the strict criteria for patient enrollment, the study has a relatively small sample size (31 VZV meningitis cases), which limited the statistical power and generalizability of the findings. Moreover, the 31 patients with VZV meningitis in this study all received active intervention treatment in the early stage, and most of them achieved good prognosis, with only four patients presenting an unfavorable prognosis at discharge, rendering the prognostic correlation findings preliminary. A larger-scale multicenter study is needed to validate these findings and to improve the generalizability and reliability of the prognostic conclusions. Secondly, the inflammatory markers were measured only at admission without dynamic observation over time, limiting understanding of their temporal relationship with disease progression. In the future, more rational prospective studies should be designed to avoid this deficiency. Thirdly, the mRS score also has certain limitations. Its evaluation method was relatively subjective and may be affected by comorbidities and the patient’s socioeconomic status. Furthermore, the mRS is mainly used to evaluate the clinical outcomes of stroke patients, and its application in the assessment of the prognosis of patients with CNS infection may have low sensitivity. However, previous scholars have used the mRS to evaluate the clinical prognosis of patients with VZV-related CNS infection, which achieved good results (Corral et al., 2020).

## 5 Conclusion

Patients with VZV meningitis exhibit significant inflammation dysfunction. The PLR has certain clinical value in the diagnosis and prognosis of VZV meningitis. Clinicians should pay attention to acute HZ patients with elevated blood PLR levels and be alert to the possibility of CNS infection. An early and timely intervention may reduce the incidence of VZV-related CNS infections and improve the quality of life of patients.

## Data Availability

The raw data supporting the conclusions of this article will be made available by the authors, without undue reservation.

## References

[B1] Akya A. Ahmadi K. Zehtabian S. Salimi A. Elahi A. Madani S. H. (2015). Study of the frequency of herpesvirus infections among patients suspected aseptic meningitis in the west of Iran. Jundishapur J. Microbiol. 8, e22639. doi: 10.5812/jjm.22639 26568804 PMC4641434

[B2] Amjadi O. Rafiei A. Haghshenas M. Navaei R. A. Valadan R. Hosseini-Khah Z.. (2017). A systematic review and meta-analysis of seroprevalence of varicella zoster virus: A nationwide population-based study. J. Clin. Virol. 87, 49–59. doi: 10.1016/j.jcv.2016.12.001 28011413

[B3] Andrei G. Snoeck R. (2021). Advances and perspectives in the management of varicella-zoster virus infections. Molecules 26, 1132. doi: 10.3390/molecules26041132 33672709 PMC7924330

[B4] Arruti M. Piñeiro L. D. Salicio Y. Cilla G. Goenaga M. A. López de Munain A. (2017). Incidence of varicella zoster virus infections of the central nervous system in the elderly: a large tertiary hospital-based serie-2014). J. Neurovirol. 23, 451–459. doi: 10.1007/s13365-017-0519-y 28224485

[B5] Becerra J. C. Sieber R. Martinetti G. Costa S. T. Meylan P. Bernasconi E. (2013). Infection of the central nervous system caused by varicella zoster virus reactivation: a retrospective case series study. Int. J. Infect. Dis. 17, e529–e534. doi: 10.1016/j.ijid.2013.01.031 23566589

[B6] Bhojwani D. Zakarya M. Abu Khalaf S. (2022). Atypical manifestation of VZV infection in a vaccinated immunocompetent adult. Case Rep. Infect. Dis. 2022, 5626670. doi: 10.1155/2022/5626670 36405549 PMC9674416

[B7] Blank L. J. Polydefkis M. J. Moore R. D. Gebo K. A. (2012). Herpes zoster among persons living with HIV in the current antiretroviral therapy era. J. Acquir. Immune Defic. Syndr. 61, 203–207. doi: 10.1097/QAI.0b013e318266cd3c 22766968 PMC4154488

[B8] Blennow O. Fjaertoft G. Winiarski J. Ljungman P. Mattsson J. Remberger M. (2014). Varicella-zoster reactivation after allogeneic stem cell transplantation without routine prophylaxis–the incidence remains high. Biol. Blood Marrow Transplant. 20, 1646–1649. doi: 10.1016/j.bbmt.2014.06.002 24914821

[B9] Carestia A. Godin L. C. Jenne C. N. (2023). Step up to the platelet: Role of platelets in inflammation and infection. Thromb. Res. 231, 182–194. doi: 10.1016/j.thromres.2022.10.001 36307228

[B10] Corral C. Quereda C. Muriel A. Martínez-Ulloa P. L. González-Gómez F. J. Corral Í. (2020). Clinical spectrum and prognosis of neurological complications of reactivated varicella-zoster infection: the role of immunosuppression. J. Neurovirol. 26, 696–703. doi: 10.1007/s13365-020-00872-x 32696182

[B11] Dolan R. D. Lim J. McSorley S. T. Horgan P. G. McMillan D. C. (2017). The role of the systemic inflammatory response in predicting outcomes in patients with operable cancer: Systematic review and meta-analysis. Sci. Rep. 7, 16717. doi: 10.1038/s41598-017-16955-5 29196718 PMC5711862

[B12] Dulin M. Chevret S. Salmona M. Jacquier H. Bercot B. Molina J. M.. (2024). New insights into the therapeutic management of varicella zoster virus meningitis: A series of 123 polymerase chain reaction-confirmed cases. Open Forum Infect. Dis. 11, ofae340. doi: 10.1093/ofid/ofae340 38957692 PMC11218771

[B13] Endo K. Satoh T. Yoshino Y. Kondo S. Kawakami Y. Katayama T.. (2021). Neutrophil-to-lymphocyte and platelet-to-lymphocyte ratios as noninvasive predictors of the therapeutic outcomes of systemic corticosteroid therapy in ulcerative colitis. Inflammation Intest Dis. 6, 218–224. doi: 10.1159/000520523 PMC8740212 35083287

[B14] Farah R. Ibrahim R. Nassar M. Najib D. Zivony Y. Eshel E. (2017). The neutrophil/lymphocyte ratio is a better addition to C-reactive protein than CD64 index as a marker for infection in COPD. Panminerva Med. 59, 203–209. doi: 10.23736/s0031-0808.17.03296-7 28185443

[B15] Forbes H. J. Bhaskaran K. Grint D. Hu V. H. Langan S. M. McDonald H. I.. (2021). Incidence of acute complications of herpes zoster among immunocompetent adults in England: a matched cohort study using routine health data. Br. J. Dermatol. 184, 1077–1084. doi: 10.1111/bjd.19687 33216946 PMC8607468

[B16] Gershon A. A. Breuer J. Cohen J. I. Cohrs R. J. Gershon M. D. Gilden D.. (2015). Varicella zoster virus infection. Nat. Rev. Dis. Primers 1, 15016. doi: 10.1038/nrdp.2015.16 27188665 PMC5381807

[B17] Giannelos N. Curran D. Nguyen C. Kagia C. Vroom N. Vroling H. (2024). The incidence of herpes zoster complications: A systematic literature review. Infect. Dis. Ther. 13, 1461–1486. doi: 10.1007/s40121-024-01002-4 38896390 PMC11219681

[B18] Grahn A. Studahl M. (2015). Varicella-zoster virus infections of the central nervous system – Prognosis, diagnostics and treatment. J. Infect. 71, 281–293. doi: 10.1016/j.jinf.2015.06.004 26073188

[B19] Harpaz R. Leung J. W. (2019). The epidemiology of herpes zoster in the United States during the era of varicella and herpes zoster vaccines: changing patterns among older adults. Clin. Infect. Dis. 69, 341–344. doi: 10.1093/cid/ciy953 30496358

[B20] Hung C. H. Chang K. H. Kuo H. C. Huang C. C. Liao M. F. Tsai Y. T.. (2012). Features of varicella zoster virus myelitis and dependence on immune status. J. Neurol. Sci. 318, 19–24. doi: 10.1016/j.jns.2012.04.017 22564884

[B21] Kazancioglu S. Bastug A. Ozbay B. O. Tezcan H. Buyuktarakci C. Akbay A.. (2023). The usefulness of hematological parameters and cerebrospinal fluid indexes in the differential diagnosis of acute bacterial from viral meningitis. Diagn. Microbiol. Infect. Dis. 107, 116005. doi: 10.1016/j.diagmicrobio.2023.116005 37392600

[B22] Kennedy P. G. Mogensen T. H. (2020). Determinants of neurological syndromes caused by varicella zoster virus (VZV). J. Neurovirol. 26, 482–495. doi: 10.1007/s13365-020-00857-w 32495195 PMC7438298

[B23] Kennedy P. G. E. Mogensen T. H. (2021). Varicella-zoster virus infection of neurons derived from neural stem cells. Viruses 13, 485. doi: 10.3390/v13030485 33804210 PMC7999439

[B24] Kim Y. Choi H. Jung S. M. Song J. J. Park Y. B. Lee S. W. (2019). Systemic immune-inflammation index could estimate the cross-sectional high activity and the poor outcomes in immunosuppressive drug-naïve patients with antineutrophil cytoplasmic antibody-associated vasculitis. Nephrol. (Carlton) 24, 711–717. doi: 10.1111/nep.13491 30203901

[B25] Le Bot A. Ballerie A. Pronier C. Bénézit F. Reizine F. Tas M.. (2021). Characteristics and outcome of varicella-zoster virus central nervous system infections in adults. Eur. J. Clin. Microbiol. Infect. Dis. 40, 2437–2442. doi: 10.1007/s10096-021-04245-y 33907935

[B26] Li Y. An Z. Yin D. Liu Y. Huang Z. Xu J.. (2016). Disease burden due to herpes zoster among population aged ≥50 years old in China: A community based retrospective survey. PloS One 11, e0152660. doi: 10.1371/journal.pone.0152660 27055179 PMC4824529

[B27] Liu H. Wang J. Zhang Y. Gu J. Wang Y. Yan Y.. (2023). Cerebrospinal fluid proteomics in meningitis patients with reactivated varicella zoster virus. Immun. Inflammation Dis. 11, e1038. doi: 10.1002/iid3.1038 PMC10549851 37904697

[B28] Lu W. H. Lin C. W. Wang C. Y. Chen L. K. Hsiao F. Y. (2018). Epidemiology and long-term disease burden of herpes zoster and postherpetic neuralgia in Taiwan: a population-based, propensity score-matched cohort study. BMC Public Health 18, 369. doi: 10.1186/s12889-018-5247-6 29554872 PMC5859733

[B29] Nagel M. A. Niemeyer C. S. Bubak A. N. (2020). Central nervous system infections produced by varicella zoster virus. Curr. Opin. Infect. Dis. 33, 273–278. doi: 10.1097/qco.0000000000000647 32332223 PMC13183292

[B30] Nassri A. Muftah M. Nassri R. Fialho A. Fialho A. Ribeiro B.. (2020). Novel inflammatory-nutritional biomarkers as predictors of histological activity in crohn’s disease. Clin. Lab. 66, 1173–1181. doi: 10.7754/Clin.Lab.2019.190816 32658409

[B31] Ozdemir R. Tuncer C. Güven A. Sezgin A. T. (2000). A case of herpes zoster misdiagnosed and treated as unstable angina pectoris. J. Eur. Acad. Dermatol. Venereol. 14, 317–319. doi: 10.1046/j.1468-3083.2000.00071-5.x 11204530

[B32] Parkes-Smith J. Chaudhuri A. (2022). Varicella zoster virus: an under-recognised cause of central nervous system infections? Intern. Med. J. 52, 100–104. doi: 10.1111/imj.15048 32896944

[B33] Ramachandran P. S. Wilson M. R. Catho G. Blanchard-Rohner G. Schiess N. Cohrs R. J.. (2021). Meningitis caused by the live varicella vaccine virus: metagenomic next generation sequencing, immunology exome sequencing and cytokine multiplex profiling. Viruses 13, 2286. doi: 10.3390/v13112286 34835092 PMC8620440

[B34] Sakurai K. Chubachi S. Irie H. Tsutsumi A. Kameyama N. Kamatani T.. (2018). Clinical utility of blood neutrophil-lymphocyte ratio in Japanese COPD patients. BMC Pulm Med. 18, 65. doi: 10.1186/s12890-018-0639-z 29720140 PMC5932787

[B35] Sauerbrei A. (2016). Diagnosis, antiviral therapy, and prophylaxis of varicella-zoster virus infections. Eur. J. Clin. Microbiol. Infect. Dis. 35, 723–734. doi: 10.1007/s10096-016-2605-0 26873382

[B36] Schmidt S. A. Kahlert J. Vestergaard M. Schønheyder H. C. Sørensen H. T. (2016). Hospital-based herpes zoster diagnoses in Denmark: rate, patient characteristics, and all-cause mortality. BMC Infect. Dis. 16, 99. doi: 10.1186/s12879-016-1369-6 26932311 PMC4773995

[B37] Soysal A. Gönüllü E. Yıldız İ. Karaböcüoğlu M. (2021). Incidence of varicella and herpes zoster after inclusion of varicella vaccine in national immunization schedule in Turkey: time trend study. Hum. Vaccin Immunother. 17, 731–737. doi: 10.1080/21645515.2020.1788861 32703071 PMC7993137

[B38] van der Willik K. D. Fani L. Rizopoulos D. Licher S. Fest J. Schagen S. B.. (2019). Balance between innate versus adaptive immune system and the risk of dementia: a population-based cohort study. J. Neuroinflammation. 16, 68. doi: 10.1186/s12974-019-1454-z 30927918 PMC6441146

[B39] van Oorschot D. Vroling H. Bunge E. Diaz-Decaro J. Curran D. Yawn B. (2021). A systematic literature review of herpes zoster incidence worldwide. Hum. Vaccin Immunother. 17, 1714–1732. doi: 10.1080/21645515.2020.1847582 33651654 PMC8115759

[B40] Werner R. N. Nikkels A. F. Marinović B. Schäfer M. Czarnecka-Operacz M. Agius A. M.. (2017). European consensus-based (S2k) Guideline on the Management of Herpes Zoster - guided by the European Dermatology Forum (EDF) in cooperation with the European Academy of Dermatology and Venereology (EADV), Part 1: Diagnosis. J. Eur. Acad. Dermatol. Venereol. 31, 9–19. doi: 10.1111/jdv.13995 27804172

[B41] Wu B. Liu G. Yube K. Ueno M. Tanaka S. Onodera M.. (2009). Effects of platelet release products on neutrophilic activity in human whole blood. Inflammation Res. 58, 321–328. doi: 10.1007/s00011-009-8230-y 19234810

[B42] Xu T. Weng Z. Pei C. Yu S. Chen Y. Guo W.. (2017). The relationship between neutrophil-to-lymphocyte ratio and diabetic peripheral neuropathy in Type 2 diabetes mellitus. Med. (Baltimore). 96, e8289. doi: 10.1097/md.0000000000008289 PMC5690705 29137012

[B43] Yan Y. Yuan Y. Wang J. Zhang Y. Liu H. Zhang Z. (2022). Meningitis/meningoencephalitis caused by varicella zoster virus reactivation: a retrospective single-center case series study. Am. J. Transl. Res. 14, 491–500. 35173869 PMC8829630

[B44] Yenikomshian M. A. Guignard A. P. Haguinet F. LaCasce A. S. Skarin A. T. Trahey A.. (2015). The epidemiology of herpes zoster and its complications in Medicare cancer patients. BMC Infect. Dis. 15, 106. doi: 10.1186/s12879-015-0810-6 25888128 PMC4352235

[B45] Zhang S. Diao J. Qi C. Jin J. Li L. Gao X.. (2018). Predictive value of neutrophil to lymphocyte ratio in patients with acute ST segment elevation myocardial infarction after percutaneous coronary intervention: a meta-analysis. BMC Cardiovasc. Disord. 18, 75. doi: 10.1186/s12872-018-0812-6 29716535 PMC5930503

[B46] Zhong J. H. Huang D. H. Chen Z. Y. (2017). Prognostic role of systemic immune-inflammation index in solid tumors: a systematic review and meta-analysis. Oncotarget 8, 75381–75388. doi: 10.18632/oncotarget.18856 29088873 PMC5650428

